# Dosimetric feasibility of stereotactic ablative radiotherapy in pulmonary vein isolation for atrial fibrillation using intensity‐modulated proton therapy

**DOI:** 10.1002/acm2.13239

**Published:** 2021-04-04

**Authors:** Xue‐Ying Ren, Peng‐Kang He, Xian‐Shu Gao, Zhi‐Lei Zhao, Bo Zhao, Yun Bai, Si‐Wei Liu, Kang Li, Shang‐Bin Qin, Ming‐Wei Ma, Jing Zhou, Yi Rong

**Affiliations:** ^1^ Department of Radiation Oncology Peking University First Hospital Beijing China; ^2^ Department of Cardiology Peking University First Hospital Beijing China; ^3^ Department of Radiation Oncology Yizhou International Proton Therapy Medical Center Hebei China; ^4^ Department of Radiation Oncology Mayo Clinic Arizona Phoenix AZ USA

**Keywords:** atrial fibrillation, helical tomotherapy, intensity‐modulated proton therapy, stereotactic ablative radiotherapy, volumetric‐modulated arc therapy

## Abstract

**Purpose:**

To evaluate dosimetric properties of intensity‐modulated proton therapy (IMPT) for simulated treatment planning in patients with atrial fibrillation (AF) targeting left atrial‐pulmonary vein junction (LA‐PVJ), in comparison with volumetric‐modulated arc therapy (VMAT) and helical tomotherapy (TOMO).

**Methods:**

Ten thoracic 4D‐CT scans with respiratory motion and one with cardiac motion were used for the study. Ten respiratory 4D‐CTs were planned with VMAT, TOMO, and IMPT for simulated AF. Targets at the LA‐PVJ were defined as wide‐area circumferential ablation line. A single fraction of 25 Gy was prescribed to all plans. The interplay effects from cardiac motion were evaluated based on the cardiac 4D‐CT scan. Dose‐volume histograms (DVHs) of the ITV and normal tissues were compared. Statistical analysis was evaluated via one‐way Repeated‐Measures ANOVA and Friedman’s test with Bonferroni’s multiple comparisons test.

**Results:**

The median volume of ITV was 8.72cc. All plans had adequate target coverage (V_23.75Gy_ ≥ 99%). Compared with VMAT and TOMO, IMPT resulted in significantly lower dose of most normal tissues. For VMAT, TOMO, and IMPT plans, D_mean_ of the whole heart was 5.52 ± 0.90 Gy, 5.89 ± 0.78 Gy, and 3.01 ± 0.57 Gy (*P* < 0.001), mean dose of pericardium was 4.74 ± 0.76 Gy, 4.98 ± 0.62 Gy, and 2.59 ± 0.44 Gy (*P* < 0.001), and D_0.03cc_ of left circumflex artery (LCX) was 13.96 ± 5.45 Gy, 14.34 ± 5.91 Gy, and 8.43 ± 7.24 Gy (*P* < 0.001), respectively. However, no significant advantage for one technique over the others was observed when examining the D_0.03cc_ of esophagus and main bronchi.

**Conclusions:**

IMPT targeting LA‐PVJ for patients with AF has high potential to reduce dose to surrounding tissues compared to VMAT or TOMO. Motion mitigation techniques are critical for a particle‐therapy approach.

## 1 INTRODUCTION

Atrial fibrillation (AF) is the most common cardiac arrhythmia^1^ mostly caused by ectopic beats initiated mostly from the pulmonary veins (PVs) entering the left atrium.^2^ A well‐established approach for treatment of symptomatic drug‐refractory patients with AF is catheter ablation (CA) by generating electrical isolation of the left atrial‐pulmonary vein junction (LA‐PVJ) which includes radiofrequency ablation and freeze ablation.^1^, ^3^ However, ablation procedures are invasive, complicated, and requires sufficient training and experience. The risk of congestive heart failure, stroke, bleeding, and complications of CA increase hospitalizations rate.^4^ The incidence of major complications with CA, including bleeding, atrioesophageal fistula, pericarditis, cardiac tamponade/perforation, and even death,^1^ is approximately 5%.^5^, ^6^, ^7^ In patients with paroxysmal and nonparoxysmal AF, the overall 1‐year recurrence rate after single procedure is about 40% or more.^8^

Stereotactic ablative radiotherapy (SABR) is a matured treatment technique that delivers precise and intense radiation dose in one or a few fractions to tumors while minimizing damage to surrounding tissues.^9^ Successful adaptation of SABR to target cardiac sarcoma has been reported.^10^ Preclinical studies exploring SABR using various forms of particle therapy for cardiac ablation have demonstrated histological changes and electrophysiological effects (voltage/potential amplitude or bidirectional block).^11^, ^12^, ^13^, ^14^, ^15^, ^16^, ^17^, ^18^ Using heavy ion‐based radioablation for healthy pigs, Lehmann and Rapp et al. demonstrated radiation‐induced fibrosis, microvascular damage, and chronic inflammation.^12^, ^17^ Successful clinical treatments for malignant ventricular tachycardia or recurrent paroxysmal AF have also been reported.^19^, ^20^, ^21^, ^22^, ^23^, ^24^, ^25^, ^26^, ^27^, ^28^, ^29^, ^30^, ^31^, ^32^ Therefore, SABR may be a novel and noninvasive approach for AF.

However, delivering one concentrated dose to the cardiac region can be challenging due to cardiac motion and its close proximity to critical structures. Compared to ventricular tachycardia, previous studies showed that SABR was considered more challenging for AF patients, and the reasons are multifold: (a) creating electrical isolation of the LA‐PVJ requires a significantly complex target; (b) it is unclear whether single fraction 25 Gy is adequate; and (c) the close proximity of esophagus and bronchus to the target may be a limiting factor.^33^, ^34^, ^35^ In addition, long‐term follow‐up in some clinical trials for patients with breast cancer has shown that radiotherapy can increase the subsequent rate of ischemic heart disease, presumably through incidental exposure of the heart to ionizing radiation.^36^ Several studies revealed that radiation dose to the heart in lung cancer patients was associated with the risk of cardiac events^37^, ^38^, ^39^, ^40^, ^41^ and overall survival.^37^, ^40^, ^42^, ^43^, ^44^, ^45^ While cardiotoxicity is usually considered as a latent event after 10 years, acute toxicity has also been observed early after radiotherapy.^36^, ^44^

Previous reports of non‐invasive ablative radiation for ventricular tachycardia or recurrent paroxysmal AF used photon‐based treatment plans, i.e., volumetric‐modulated arc therapy (VMAT) or Cyberknife.^19^, ^20^, ^21^, ^22^, ^32^ It has been shown that single‐fraction ablative radiotherapy significantly reduced burden of AF and ventricular tachycardia, but some cardiac complications, i.e., pericarditis and heart failure exacerbation, have also occurred early upon treatment completion.^20^ As opposed to photon‐delivery techniques, intensity‐modulated proton therapy (IMPT) takes advantage of proton beam’s unique Bragg Peak property and high‐dose conformity with pencil beam modulation, thus may be a promising alternative for AF patients. However, IMPT is highly sensitive to tissue motion, resulting in interplay effects between treatment beams and intrafractional target motion, which could produce dosimetric cold or hot spots.^46^ Hohmann et al. reported possible decrease in left ventricular function after proton beam therapy in 20 swine and the adverse effect was dose dependent.^47^ This study aimed to investigate dosimetric properties of IMPT for non‐invasive AF ablation in comparison with photon‐base treatment techniques (including VMAT and TOMO) and the interplay effects in IMPT plan from cardiac motion.

## 2 METHODS

### 2.1 CT simulation

The study has been reviewed and approved by the institutional review board (No. 2019[240]). We retrospectively collected 11 thoracic 4D computed tomography (CT) scans from our institution, of which 10 were stamped with respiratory motion phases (patients 1–10) and 1 was stamped with cardiac motion phases (patient 11). All had received 2 thoracic 4D‐CT scans, first without and with intravenous contrast immediately thereafter. The thoracic respiratory 4D‐CT images were taken on a 16‐slice Brilliance Big Bore CT (Philips Medical Systems, Cleveland, OH, USA) and respiratory motion was traced by a pressure sensor belt. Each 4D‐CT image consists of 10 phases 3D‐CT datasets equally divided 10% of breathing phases in a complete respiratory cycle. Cardiac‐gated 4D‐CT images were taken on a 64‐slice scanner (GE Healthcare, Waukesha, USA) at the end‐expiration breath‐hold using retrospective cardiac gating and also reconstructed 10 phases of cardiac cycle. For all patients, CT simulation was performed at 3‐mm intervals.

### 2.2 Delineation of targets and organs at risk (OARs)

**Fig. 1:**
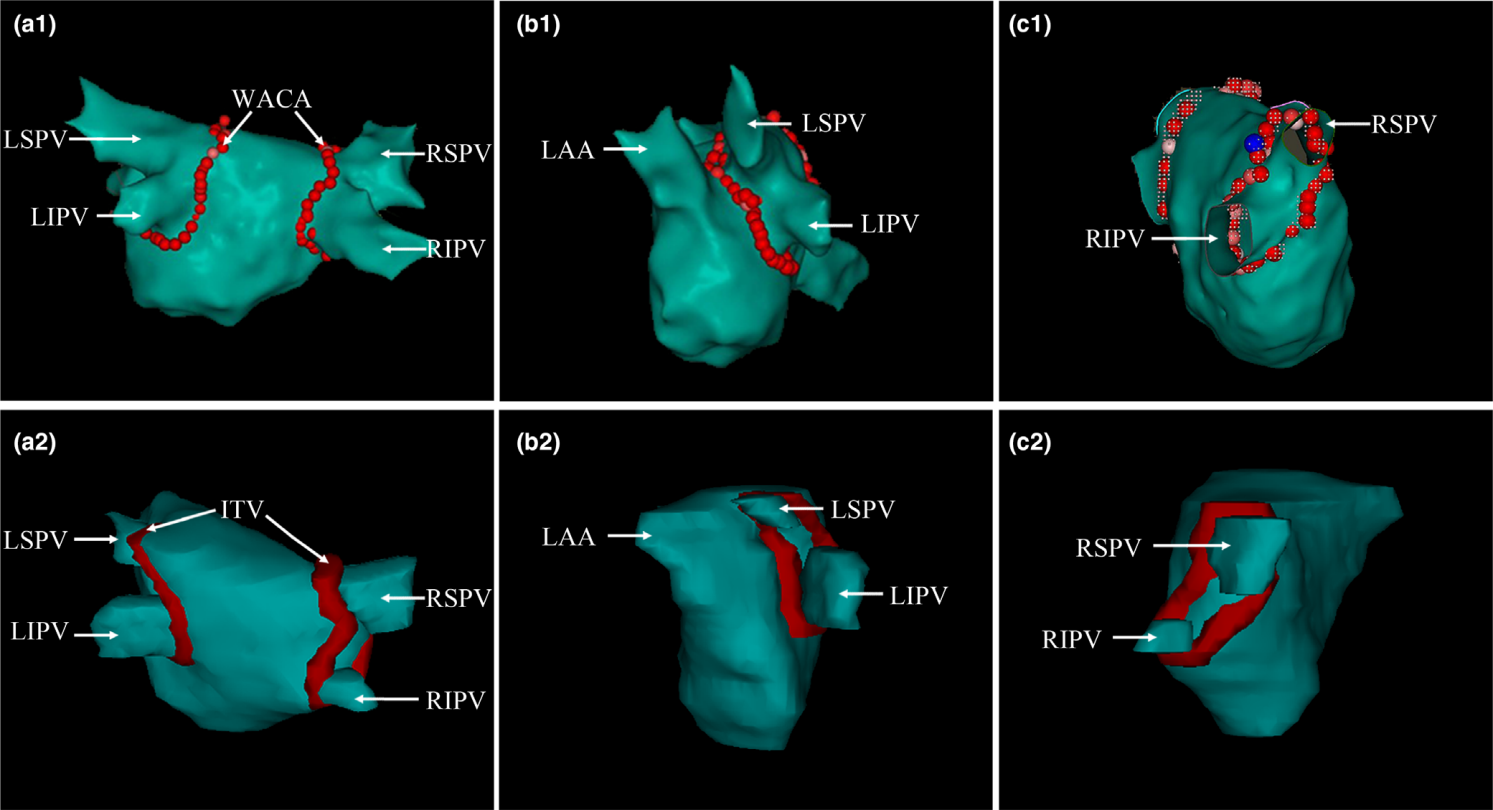
Volume of wide‐area circumferential ablation (a1‐c1) around the PVs that was created by the CARTO 3 system (Biosense‐Webster, Diamond Bar, CA, USA) on postero‐anterior, left‐lateral, and right‐lateral sight. Volume of ITV (a2‐c2) on the same sight of the other person in this study, respectively. *Abbreviations:* LSPV = left superior pulmonary vein, LIPV = left inferior pulmonary vein, RSPV = right superior pulmonary vein, RIPV = right inferior pulmonary vein, LAA = left atrial appendage

The clinical target volume (CTV) was created aiming at LA‐PVJ as wide‐area circumferential ablation line (WACA) on each phase and generated 3D clinical target volumes ensuring continuity, including left CTV (CTVL) and right CTV (CTVR). WACA was the routine volume in catheter ablation for AF. The ITV was generated from combining CTVs from 10 phases in view of maximum range of respiratory or cardiac motion. The volumes of CTV and ITV were shown in Figure 1. No gross tumor volume (GTV) was utilized.

All normal structures were contoured on averaged intensity projection (AIP) of 4D‐CT scans according to RTOG 1106 OARs contouring atlas for thoracic radiotherapy,^48^ including esophagus, spinal cord, total lung, main bronchi, whole heart, pericardium, four great vessels (pulmonary artery, aorta, superior vena, and inferior vena cava), and eight cardiac substructures. These were also contoured on all phases of the cardiac‐gated 4D‐CT.

Cardiac substructures covered four cardiac chambers (atria and ventricles) and four coronary arteries (left main coronary artery [LMC], left anterior descending artery [LAD], left circumflex artery [LCX], and right coronary artery [RCA]). All cardiac substructures were outlined according to previous published cardiac atlas. ^49^, ^50^ All the targets and OARs were contoured by one experienced radiation oncologist and evaluated by one senior cardiac electrophysiologist.

### 2.3 Treatment planning

The AIP dataset was used to create all photon and proton plans for 10 patients about respiratory motion. A single fraction of 25 Gy was used as the prescription for all plans. For the photon plans, PTV was expanded uniformly 3 mm from ITV and physical dose of 25 Gy was prescribed to the PTV; while a total dose of 25 GyE (relative biological effectiveness 1.1) was prescribed to ITV for the proton plans. Normal tissue constraints include 0.03 cc of spinal cord, esophagus, and main bronchi receiving no more than 10 Gy, 16 Gy, and 20.2 Gy, respectively, and total lung V_20_ ≤ 10%, V_2.5_ ≤ 50%, and V_7 Gy_ < 1500 cc. The dose to the sensitive structures was minimized, with the following priorities settings (high to low): 1 = spinal cord, 2 = esophagus, 3 = main bronchi, and 4 = total lung. Despite no hard constraints for cardiac dose exposures, we try the best to minimize dose of whole heart, pericardium, and all cardiac substructures.

All VMAT, TOMO, and IMPT plans were created blindly without prior knowledge to the quality of others. All three plans were reviewed by senior physicists and approved by one attending physician based on target goals and normal tissue constraints. VMAT plans were generated using double‐full arcs on Eclipse treatment planning system (Eclipse, Version 13.5, Varian Medical Systems, Palo Alto, CA). For the TOMO plans, a field width of 1.048 cm, pitch of 0.287, and modulation factor of 3.0 were used on the HD^TM^ treatment planning system (Version 5.1.1.6, Accuray, Sunnyvale, CA). IMPT plans were created using four beams based on IBA Proteus^®^PLUS machine model using pencil beam scanning. The dose distribution was calculated by the Monte Carlo algorithm on RayStation treatment planning system (Version 7.0, RaySearch Laboratories, Stockholm, Sweden), with robust optimization to cover 99% of ITV. Set‐up error was determined at 3 mm, and the range uncertainty at 3.5% in addition to a distal margin of 2 mm.^51^ The spot size sigma varies between 2.6 and 7.2 mm in the energy range 70–230 MeV.

When evaluating interplay effects in IMPT plans, we calculated an IMPT nominal plan with the same optimized approach as mentioned above. A “dynamic dose” was calculated using in‐house Python scripts in RayStation for evaluating interplay effects from cardiac motion. The calculation parameters used for the IMPT nominal plan included: (a) patient cardiac cycle 1.0 s; (b) spot delivery time 4.0ms/MU; (c) spot motion speed 250 cm/s; (d) energy layer shift time 2.0 s; and (v) starting phase T_00_ (0% phase of cardiac cycle). The final dose distribution was accumulated on the AIP CT images from 10 cardiac motion phases with equal weighting using deformable image registration algorithm in RayStation system.

### 2.4 Evaluation

For evaluating target displacements of cardiac motion, the maximal displacements for centroids of CTVL and CTVR at all phases of cardiac‐gated 4D‐CT were recorded in medial‐lateral (ML), anterior‐posterior (AP), and superior‐inferior (SI) direction, in reference to phase T_00_.

For dosimetric evaluation, dose‐volume histograms (DVHs) and dose distributions were calculated for ITV and other critical structures, such as the whole heart, pericardium, cardiac substructures, total lung, esophagus, main bronchi, and spinal cord. For pair comparison, all plans were normalized to 100% of prescription dose (25GyE) covering 99% ITV.

### 2.5 Statistical analysis

Data distribution was analyzed using Shapiro‐Wilk test. One‐way Repeated‐Measures ANOVA with Bonferroni’s multiple comparisons test was performed for normal distribution data. Data showing non‐normal distribution was compared using Friedman’s test with Bonferroni’s multiple comparisons test. A p value of 0.05 or less was considered to indicate statistical significance. SPSS 24.0 statistical software (IBM SPSS Inc., Armonk, NY) was used for statistical analyses.

## 3 RESULTS

### 3.1 Treatment planning study for respiratory motion

**Fig. 2:**
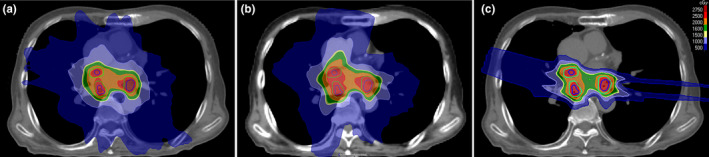
An example of dose distribution of VMAT (a), TOMO (b), and IMPT (c) for the same patient. The green lines and inner dark‐blue lines outlined PTV and ITV

For patients 1–10, median volumes of CTV, ITV, and PTV were 2.62 cc (min: 1.71 cc, max: 3.37 cc), 8.72 cc (min: 5.36 cc, max: 12.60 cc), and 30.16 cc (min: 20.53 cc, max: 36.44 cc), respectively. The median ratio of CTV to ITV was 32% (min: 21%, max: 55%). All plans provided adequate target coverage (V_23.75 Gy_ ≥ 99%). ITV V_23.75 Gy_ (%) was 99.92 ± 0.13, 99.86 ± 0.19, and 99.70 ± 0.20 for VMAT, TOMO, and IMPT plans, respectively. The dose distribution for one case is shown in Fig. 2.

As for normal tissues, dosimetric parameters of three modalities for critical structures (including total lung, spinal cord, esophagus, and main bronchi) are given in Table 1. DVH analysis showed significantly lower mean dose and V_5_ to the total lung with the IMPT plans, compared with VMAT and TOMO plans. For IMPT plans, mean dose, D_5cc_ to the esophagus, and maximum dose to the spinal cord were significantly reduced by 0.94 Gy, 2.29 Gy, and 3.03 Gy, respectively, compared with VMAT. However, D_0.03cc_ of esophagus (~14.9 Gy) and main bronchi (~11.3 Gy) was comparable in all three plans.

**Table 1:** DVH parameters (Mean ± SD) of the adjacent normal tissues

Parameters		VMAT	TOMO	IMPT	*p*1(VMAT vs. IMPT)	*p*2(TOMO vs. IMPT)	*p*3(VMAT vs. TOMO)
Total Lung	D_mean_(Gy)	2.31 ± 0.54	2.27 ± 0.41	1.26 ± 0.38	<0.001	<0.001	1.000
	V_5_ (%)	15.08 ± 6.17	14.77 ± 4.23	9.65 ± 2.92	0.005	<0.001	1.000
	V_20_ (%)	0.10 ± 0.05	0.27 ± 0.13	0.41 ± 0.35	0.001	1.000	0.005
Spinal Cord	D_0.03cc_(Gy)	8.25 ± 1.81	6.64 ± 0.78	5.22 ± 1.29	<0.001	0.076	0.076
Esophagus	D_mean_ (Gy)	2.40 ± 0.38	1.98 ± 0.46	1.46 ± 0.44	<0.001	0.076	0.076
	D_0.03cc_(Gy)^a^	14.33 ± 0.37	14.93 ± 0.83	14.88 ± 0.90			
	D_5cc_ (Gy)	4.79 ± 1.66	3.49 ± 1.83	2.50 ± 1.45	<0.001	0.221	0.042
Main Bronchi	D_0.03cc_(Gy)^a^	10.41 ± 4.64	11.79 ± 4.07	11.34 ± 4.77			

V_m_ (%) means the percentage of volume receiving at least m (Gy) doses. D_n_ (Gy) means the maximum dose received by n % volume.

Abbreviations: IMPT, intensity‐modulated proton therapy; ITV, internal target volume; TOMO, helical tomotherapy; VMAT, volumetric‐modulated arc therapy.

^a^ There was no significant difference of D_0.03cc_ to esophagus and main brochi among three groups, so we did not do paired test.

The dosimetric parameters for the rest of unconventional critical structures (including whole heart, pericardium, individual cardiac chambers, and four main coronary arteries (LMC, LAD, LCX, and RCA)) are shown in Table 2. Compared with VMAT or TOMO, IMPT improved D_mean_, V_2_, V_5_, and V_10_ of the whole heart and pericardium. Compared with VMAT and TOMO plans, IMPT reduced D_mean_ and V_5_ of the whole heart by 2.51 Gy and 2.88 Gy, 23.65% and 23.15%, respectively, which also reduced D_mean_ and V_5_ of pericardium by 2.15 Gy and 2.39 Gy, 18.94% and 18.91%, respectively. In addition, For IMPT plans, D_mean_ of four cardiac chambers was significantly lower than VMAT and TOMO plans.

**Table 2:** DVH parameters (Mean ± SD) of heart and cardiac substructures

Parameters		VMAT	TOMO	IMPT	*p*1(VMAT vs. IMPT)	*p*2(TOMO vs. IMPT)	*p*3(VMAT vs. TOMO)
Whole Heart	D_mean_(Gy)	5.52 ± 0.90	5.89 ± 0.78	3.01 ± 0.57	<0.001	<0.001	0.160
	V_2_ (%)	54.23 ± 10.83	55.69 ± 9.82	20.14 ± 3.05	<0.001	<0.001	0.435
	V_5_ (%)	39.16 ± 7.43	38.66 ± 5.68	15.51 ± 2.22	<0.001	<0.001	1.000
	V_10_ (%)	17.52 ± 3.05	20.72 ± 2.99	11.70 ± 1.82	<0.001	<0.001	0.034
	V_15_ (%)	10.14 ± 1.74	12.13 ± 2.06	9.05 ± 1.78	0.073	0.001	0.006
	V_20_ (%)	6.50 ± 1.31	7.96 ± 1.20	6.67 ± 1.78	1.000	0.038	<0.001
Pericardium	D_mean_(Gy)	4.74 ± 0.76	4.98 ± 0.62	2.59 ± 0.44	<0.001	<0.001	0.330
	V_2_ (%)	47.36 ± 8.94	47.86 ± 7.38	18.21 ± 2.41	<0.001	<0.001	1.000
	V_5_ (%)	32.46 ± 6.12	32.43 ± 4.78	13.52 ± 1.78	<0.001	<0.001	1.000
	V_10_ (%)*	14.25 ± 2.74	16.91 ± 2.64	9.85 ± 1.43	0.011	<0.001	0.035
	V_15_ (%)	8.13 ± 1.43	9.77 ± 1.61	7.49 ± 1.40	0.231	0.001	0.006
	V_20_ (%)	5.17 ± 0.98	6.34 ± 0.85	5.43 ± 1.37	0.983	0.062	<0.001
LA	D_mean_(Gy)	16.80 ± 2.26	17.48 ± 1.92	14.47 ± 2.86	0.004	0.002	0.265
LV	D_mean_(Gy)	2.14 ± 1.22	1.84 ± 0.80	0.15 ± 0.13	0.001	<0.001	0.597
RA	D_mean_(Gy)	4.84 ± 2.11	5.58 ± 2.25	2.47 ± 0.93	0.004	0.001	0.041
RV	D_mean_(Gy)	1.58 ± 0.70	1.84 ± 0.58	0.01 ± 0.00	0.022	<0.001	0.539
LMC	D_mean_(Gy)	8.44 ± 4.28	7.30 ± 2.83	0.95 ± 0.75	0.001	0.005	1.000
	D_0.03cc_(Gy)	9.90 ± 4.29	10.21 ± 3.54	1.87 ± 1.42	0.011	<0.001	1.000
LAD	D_mean_(Gy)	3.92 ± 1.47	2.75 ± 0.88	0.19 ± 0.23	<0.001	0.022	0.539
	D_0.03cc_(Gy)	8.43 ± 2.37	6.77 ± 2.21	1.52 ± 1.89	<0.001	0.011	1.000
LCX	D_mean_(Gy)	9.93 ± 4.48	7.57 ± 2.04	2.74 ± 1.81	<0.001	0.042	0.221
	D_0.03cc_(Gy)	13.96 ± 5.45	14.34 ± 5.91	8.43 ± 7.24	0.011	0.042	1.000
RCA	D_mean_(Gy)	3.87 ± 1.89	2.85 ± 1.25	0.04 ± 0.04	<0.001	0.022	0.539
	D_0.03cc_(Gy)	5.18 ± 2.06	4.63 ± 1.52	0.08 ± 0.10	<0.001	0.011	1.000

Abbreviations: IMPT, intensity‐modulated proton therapy; LA, left atrium; LAD, left anterior descending artery; LCX, left circumflex artery; LMC, left main coronary artery; LV, left ventricle; RA, right atrium; RCA, right coronary artery; RV, right ventricle; TOMO, helical tomotherapy; VMAT, volumetric‐modulated arc therapy.

**Fig. 3:**
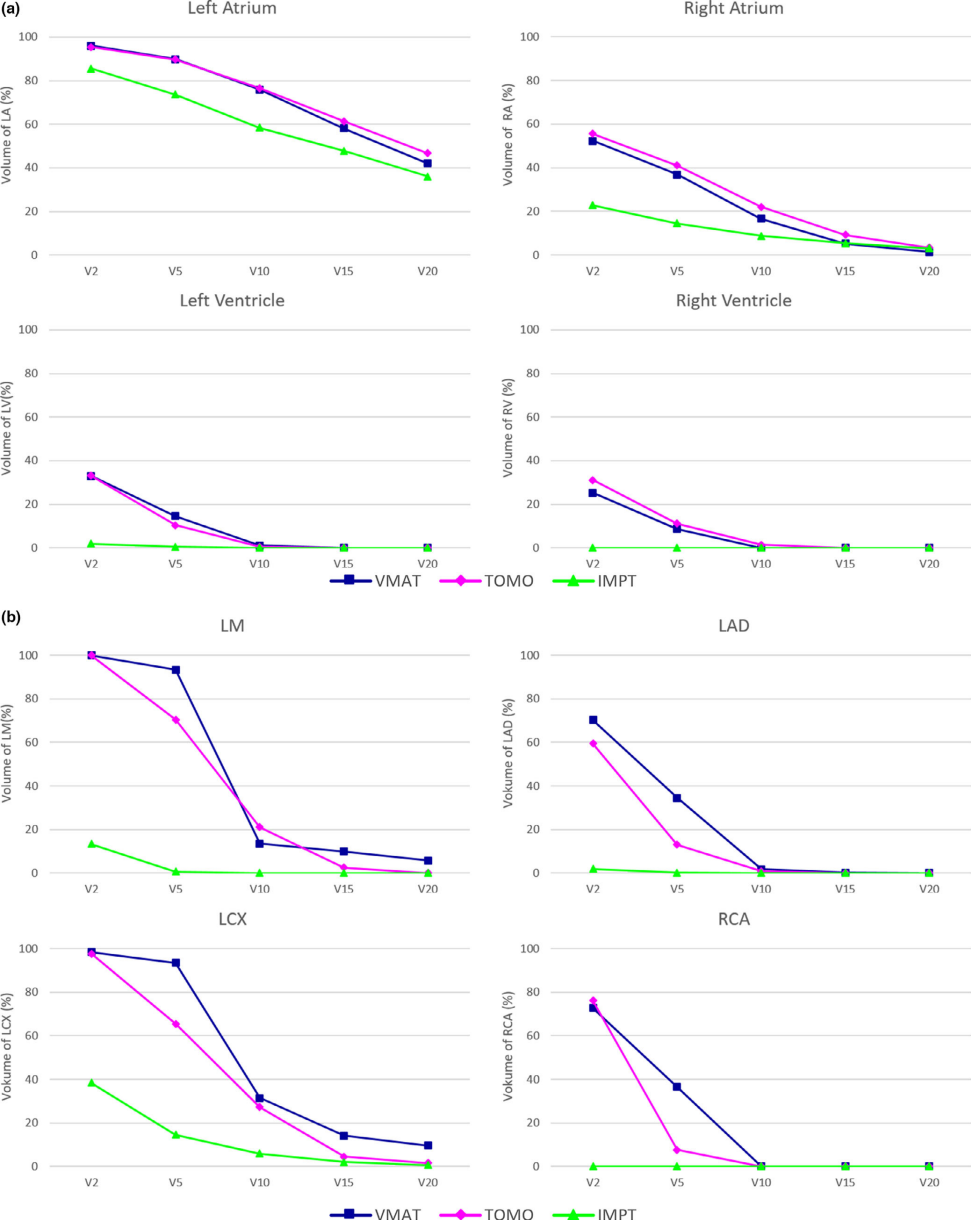
Dosimetric parameters for four cardiac chambers (a) and four main coronary arteries (b) according to radiation treatment modalities. All data are shown as mean

Dose‐volume histograms analysis of V_2_, V_5_, V_10_, V_15_, and V_20_ of four cardiac chambers and four main coronary arteries was shown in Fig. 3. When comparing IMPT with VMAT and TOMO plans, the difference in V_5_ for left ventricle was 14.11% and 10.01%; and the differences in D_0.03cc_ for LMC, LAD, LCX, and RCA were 8.03 Gy and 8.34 Gy, 6.91 Gy and 5.25 Gy, 5.53 Gy and 5.91 Gy, and 5.10 Gy and 4.55 Gy, respectively.

### 3.2 3.B Treatment planning study for cardiac motion

**Fig. 4:**
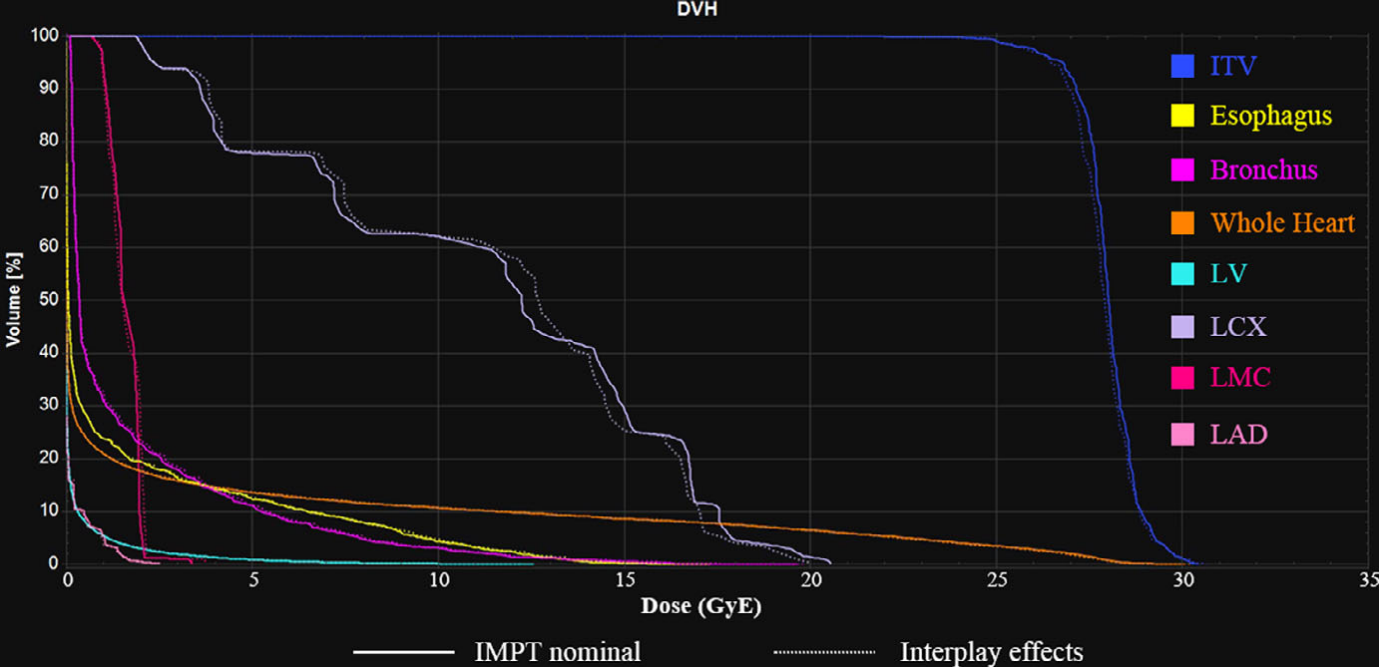
Dose‐volume histograms comparison between intensity‐modulated proton therapy nominal plan and dynamic dose considering interplay effects for patient 11. Abbreviations*:* LV = left ventricle, LCX = left circumflex artery, LMC = left main coronary artery, LAD = left anterior descending artery

For patient 11, the maximal displacements of CTVL in ML, AP, and SI directions due to cardiac motion were 2.0, 1.0, and 2.4 mm, and the maximal displacements of CTVR were 0.7, 0.5, and 1.2 mm, respectively. DVHs comparisons between IMPT nominal plan and dynamic dose considering interplay effects were shown in Fig. 4. V_23.75 Gy_ and homogeneity index (HI = D_5%_/D_95%_) of ITV in IMPT nominal plan and interplay effects were 99.80% and 99.83%, 1.09 and 1.10, respectively. For this patient, the impact of interplay effects on OARs doses was almost negligible for the robustly optimized IMPT plan.

## 4 DISCUSSION

To our knowledge, this study is the first treatment planning investigation to compare detailed dose distribution using proton beams, as compared with photon plans, for targeting at LA‐PVJ with the catheter‐free procedures to ablate AF. It demonstrates that all plans can achieve adequate target coverage for precise circumferential and electrical isolation of the LA‐PVJ. Our findings also show that compared with VMAT and TOMO, IMPT improved the sparing of normal cardiac structures at most of dose level, not including the maximum dose of esophagus and main bronchi.

Stereotactic ablative radiotherapy may be a viable option to overcome some shortcomings of previous treatment for AF ablation. In the past decades, it is very difficult to balance the safety and efficacy of radiofrequency ablation for AF. High recurrence risk of AF after CA is usually caused by inaccessible atrial myocardium and discontinuous ablation lines due to insufficient energy to generate transmural myocardial scar considering severe complications such as atrioesophageal fistula.^52^ Compared with conventional radiofrequency ablation, stereotactic arrhythmia radioablation could provide continuous ablation lines and adequate energy to generate transmural lesions for isolating electrical conduction between pulmonary veins and left atrium.

Intensity‐modulated proton therapy can reduce the dose of heart and cardiac substructures and may reduce potential adverse effects. The recent phase I/II trial reported that two patients (10.5%) who received stereotactic ablative radiotherapy (IMRT or VMAT) for malignant ventricular tachycardia experienced a grade 3 treatment‐related severe adverse effect.^20^ One patient experienced heart failure exacerbation (grade 3), and another patient had pericarditis and was treated with prednisone. These adverse events could be associated with dose exposure to the normal heart and pericardium, but it is necessary to notice that the health condition of these patients is very poor. The following secondary analysis for this trial found that the correlation between larger target volume and shorter overall survival.^19^ Compared with the radioablation of ventricular arrhythmias, which has been carried out in clinics, the targets in AF have high complexity, and close to critical structures. Hence, special attention should be paid to the dose of whole heart, pericardium, and cardiac substructures. A recent research found that rates of major coronary events (MCE) increased linearly with the mean dose to the heart by 7.4% per gray, with no apparent threshold.^36^ A validation and modification of a prediction model for acute cardiac events in patients with breast cancer treated with radiotherapy showed that left ventricle volume receiving 5 Gy (LV‐V_5_) was the most important prognostic dose‐volume parameter.^53^ For lung cancer patients underwent radiotherapy, a study showed that the rates of grade ≥3 cardiac events increased with mean heart dose by 7% per Gy.^40^ In our study, IMPT had the reduction of mean dose of whole heart by approximately 2.7 Gy and the LV‐V_5_ by at least 10% compared with those photon plans. Another study found that the risk of pericardial effusion (PCE) was related to several cardiac parameters (e.g., mean dose of whole heart / pericardium, heart / pericardium V5), and PCE rate increased with mean dose of pericardium by 5% per Gy.^37^ Our study showed decreased mean dose of pericardium, heart V_5_, and pericardium V_5_ by about 2.3 Gy, 23% and 19%, respectively, with IMPT.

Previous study found that mean dose, V_5_ and V_30_ to LAD were related to acute coronary syndrome or congestive heart failure after conventional‐dose chemoradiation therapy for lung cancer.^54^ Another study showed that coronary artery‐based model, including LAD‐V_5_ and LCX‐V_20_, was superior to the whole heart when analyzing late ischemic cardiotoxicity.^55^ In our study, IMPT significantly reduced doses to the four main coronary arteries. Except for the LCX, the mean dose of main coronary arteries was almost zero and the maximum dose was no more than 2.0 Gy. Compared with VMAT and TOMO plans, IMPT reduced D_mean_, D_0.03cc_, and V_5_ of LCX by 7.19 Gy and 4.83 Gy, 5.53 Gy and 5.91 Gy, 78.85% and 50.83%, respectively. Nevertheless, there were currently no exact dose constraints for coronary arteries, partly due to limited studies on the relationship between dose of coronary arteries and ischemic heart diseases. Therefore, IMPT has its own safety advantages in the application of atrial arrhythmia ablation, which needs further preclinical and clinical evidence to support.

For CA in AF patients, the esophageal injury is also one of the most important complications and even lethal.^1^ This study also showed that esophageal toxicity might still need special attention with IMPT. The average D_5cc_ of esophagus was 2.5 Gy and D_0.03cc_ was about 15 Gy, both of which met tolerance thresholds in thoracic single‐fraction SABR.^56^, ^57^ However, D_0.03cc_ of esophagus was not significantly improved by IMPT, due to its close proximity to targets.

In addition, motion management is challenging, especially for IMPT. When considering that patients would be treated under respiratory gated or free breathing, we used the respiratory‐phased binned 4D‐CT for dosimetric comparison among three modalities in our study, which is the same as previous clinical studies.^19^, ^20^, ^21^ In our study, we found that the median ratio of CTV to ITV is 32%. Therefore, ITV might be reduced by 68% if respiratory‐gated treatment is applied. The previous work found that displacements of LA‐PVJ were mainly affected by respiratory motion, and the mean displacements were 1–4 mm caused by cardiac contraction under breath‐hold conditions.^58^ A recent publication reported that the motion of cardiac substructures is not uniform and patient specific.^59^ Even with appropriately compensated respiration motion using various techniques, including gating, tracking, and/or breath‐holding, cardiac motion‐induced anatomy deformation is complex, which could be partially compensated using personalized margin determined based on cardiac 4D‐CT images. Interplay effects of scanned particle beams and moving targets can severely impact dose distributions.^60^, ^61^ This is a feasibility study using cardiac motion scan from one patient case to study interplay effects. More patient cases are being prospectively collected for further studying this effect. Furthermore, the interplay effects were influenced by even more complex factors involving motion amplitude, length of cardiac cycle, starting phase, number of spots, and others.^61^, ^62^ Thus, it also needs further investigation based on more complex patterns of cardiac movement, randomized starting phases, and motion amplitude. If the interplay effects need to be mitigated further, beam rescanning and 4D dose calculation may be optional. Constantinescu et al. reported that 10 or more rescans might be adequate to mitigate motion from cardiac contraction.^61^

There are also other limitations in this study. The simulated irradiation volumes in normal cardiac images were used, instead of AF patients who usually had a nonconstant cyclical heartbeat, cardiac insufficiency, and enlarged left atrium. In addition, when implementing IMPT for animal experiments or patients with AF, there are some critical aspects that need more attention, which is not considered in this dosimetric study. Although some studies showed that 25 Gy in a single fraction could create electrical block and fibrosis for AF in animals,^11^, ^15^, ^16^ others reported that much higher doses are needed (>25 Gy) to create therapeutic effects and transmural scar.^12^, ^13^, ^14^ Furthermore, there could be room for dosimetric improvement at a potential sacrifice of overall longer treatment time, although we tried to achieve the best optimal plans of each type during planning in this study. For example, VMAT plan quality might be marginally improved with more noncoplanar partial arcs, instead of the 2‐full‐arc VMAT plans used in the presented comparison. This current study is limited without further studying those planning options among the same delivery modality. All above‐mentioned issues require further mathematical modelling, phantom, and clinical evaluation, which is being investigated in our future studies.

## 5 Conclusions

IMPT has high potential to reduce dose to surrounding tissues due to its sharp dose gradients but motion mitigation techniques are essential for a particle therapy approach for AF. This study sheds light on the use of proton therapy for noninvasive radiotherapy for AF.

## CONFLICTS OF INTEREST

None.

## AUTHOR’S CONTRIBUTIONS

Conception and design: XYR, PKH, XSG, YR, and JZ. Acquisition of data: XYR, ZLZ, BZ, YB, SWL, KL, SBQ, and MWM. Analysis of data: XYR. Writing the first draft of the manuscript: XYR and PKH. All authors contributed to the drafting and editing of the manuscript and approved the final version.

## Data Availability

The data that support the findings of this study are available from the corresponding author upon reasonable request.
